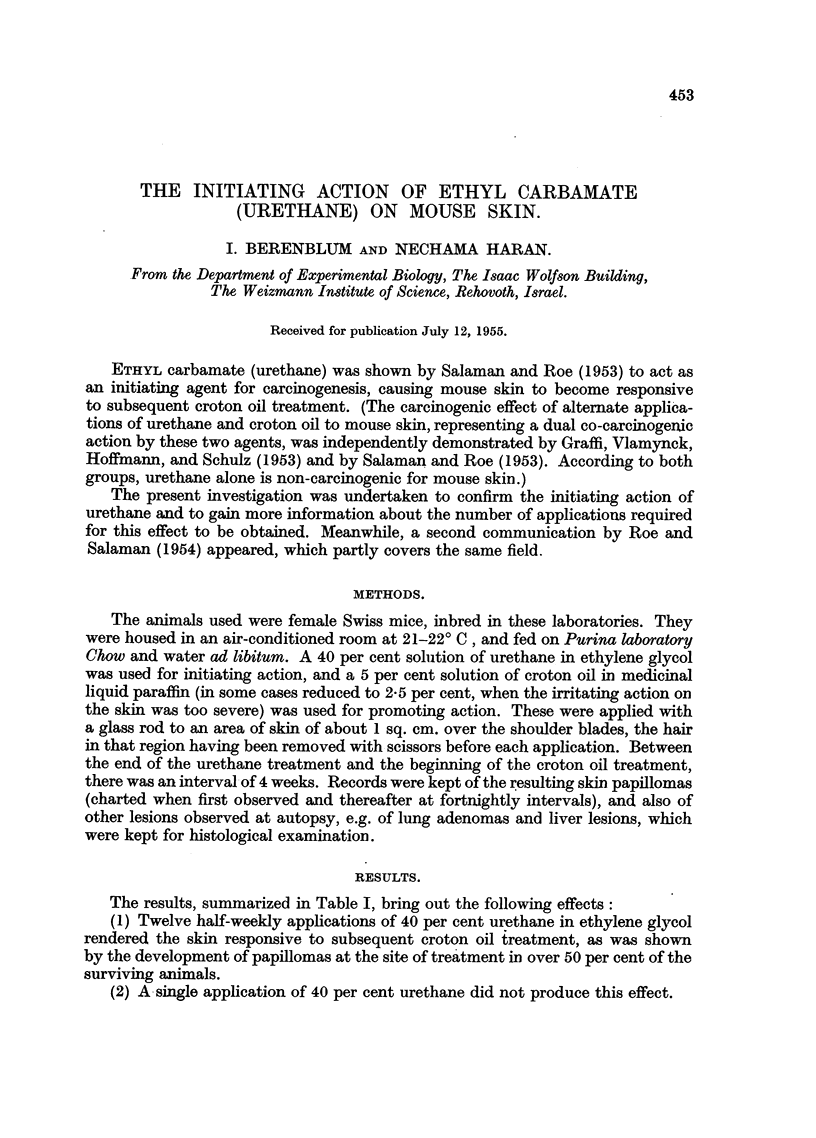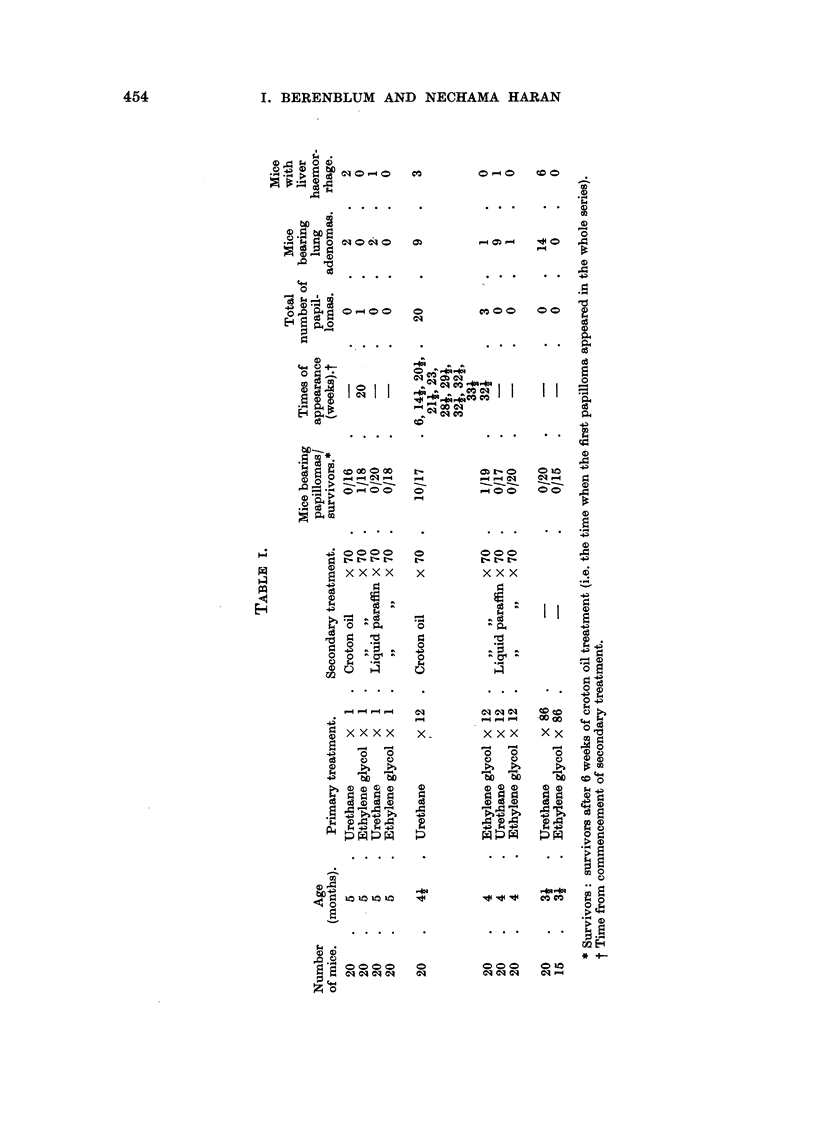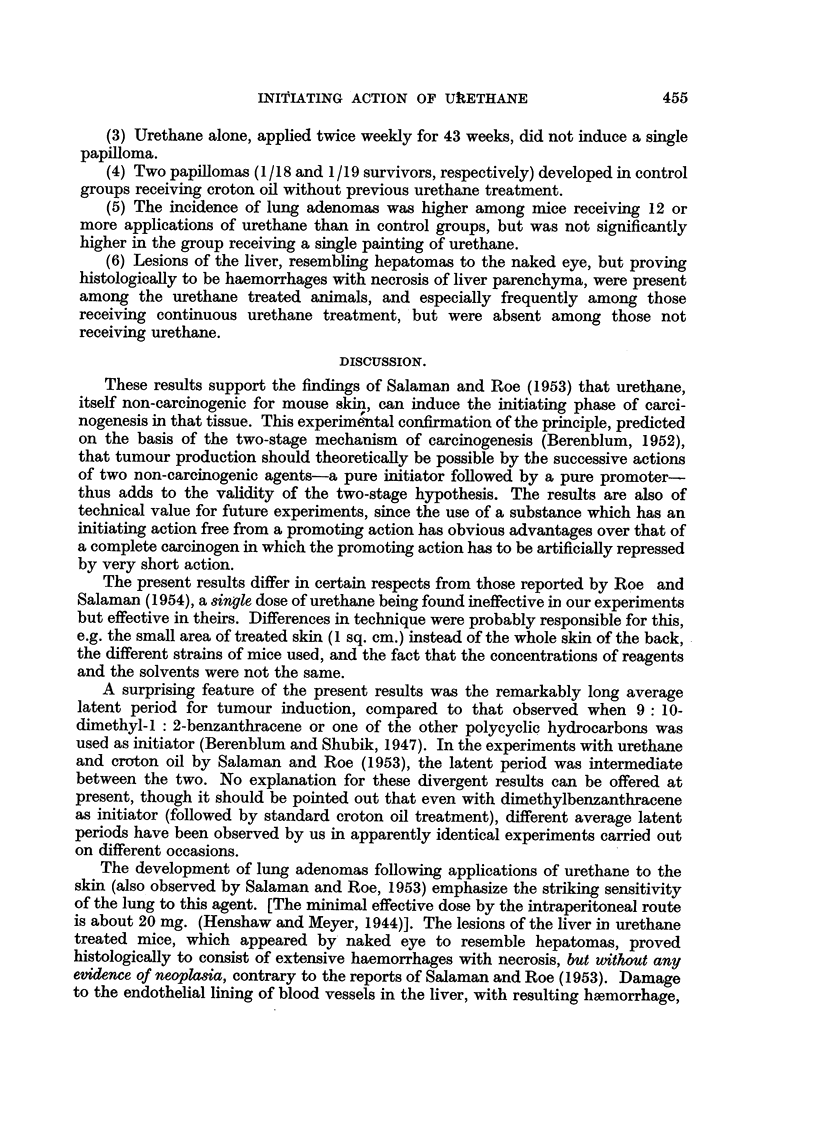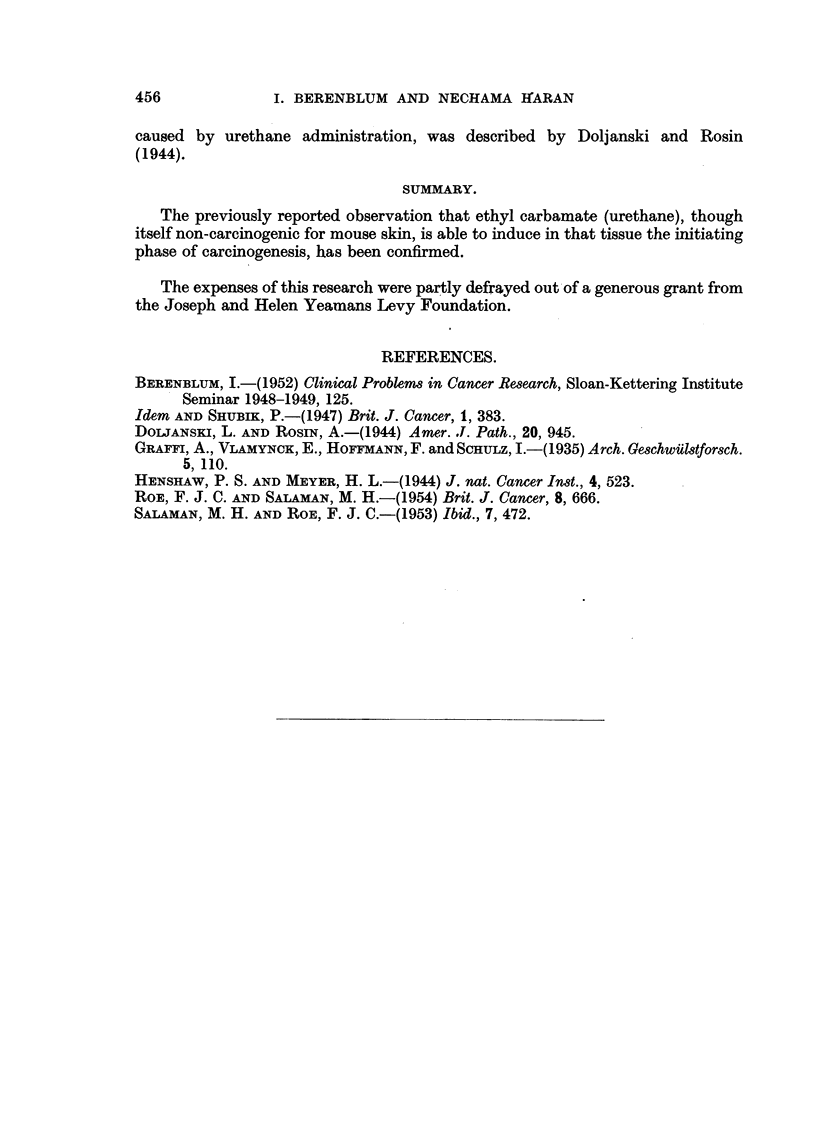# The Initiating Action of Ethyl Carbamate (Urethane) on Mouse Skin

**DOI:** 10.1038/bjc.1955.45

**Published:** 1955-09

**Authors:** I. Berenblum, Nechama Haran


### THE INITIATING ACTION OF ETHYL CARBAMATE (URETHANE) ON MOUSE SKIN.

I. BERENBLUM AND NECHAMA HARAN.

*From the Department of Experimental Biology, The Isaac Wolfson Building, The Weizmann Institute of Science, Rehovoth, Israel.*

Received for publication July 12, 1955.

ETHYL carbamate (urethane) was shown by Salaman and Roe (1953) to act as an initiating agent for carcinogenesis, causing mouse skin to become responsive to subsequent croton oil treatment. (The carcinogenic effect of alternate applications of urethane and croton oil to mouse skin, representing a dual co-carcinogenic action by these two agents, was independently demonstrated by Graffi, Vlamynck, Hoffmann, and Schulz (1953) and by Salaman and Roe (1953). According to both groups, urethane alone is non-carcinogenic for mouse skin.)

The present investigation was undertaken to confirm the initiating action of urethane and to gain more information about the number of applications required for this effect to be obtained. Meanwhile, a second communication by Roe and Salaman (1954) appeared, which partly covers the same field.

### METHODS.

The animals used were female Swiss mice, inbred in these laboratories. They were housed in an air-conditioned room at 21–22° C , and fed on *Purina laboratory Chow* and water *ad libitum.* A 40 per cent solution of urethane in ethylene glycol was used for initiating action, and a 5 per cent solution of croton oil in medicinal liquid paraffin (in some cases reduced to 2·5 per cent, when the irritating action on the skin was too severe) was used for promoting action. These were applied with a glass rod to an area of skin of about 1 sq. cm. over the shoulder blades, the hair in that region having been removed with scissors before each application. Between the end of the urethane treatment and the beginning of the croton oil treatment, there was an interval of 4 weeks. Records were kept of the resulting skin papillomas (charted when first observed and thereafter at fortnightly intervals), and also of other lesions observed at autopsy, e.g. of lung adenomas and liver lesions, which were kept for histological examination.

### RESULTS.

The results, summarized in Table I, bring out the following effects :

(1) Twelve half-weekly applications of 40 per cent urethane in ethylene glycol rendered the skin responsive to subsequent croton oil treatment, as was shown by the development of papillomas at the site of treatment in over 50 per cent of the surviving animals.

(2) A single application of 40 per cent urethane did not produce this effect.

### TABLE I.

| Number of mice. | Age (months). | Primary treatment. | Secondary treatment. | Mice bearing papillomas/ survivors.* | Times of appearance (weeks).† | Total number of papil-lomas. | Mice bearing lung adenomas. | Mice with liver haemor-rhage. |
|---|---|---|---|---|---|---|---|---|
| 20 | 5 | Urethane × 1 | Croton oil × 70 | 0/16 | — | 0 | 2 | 2 |
| 20 | 5 | Ethylene glycol × 1 | „ „ × 70 | 1/18 | 20 | 1 | 0 | 0 |
| 20 | 5 | Urethane × 1 | Liquid paraffin × 70 | 0/20 | — | 0 | 2 | 1 |
| 20 | 5 | Ethylene glycol × 1 | „ „ × 70 | 0/18 | — | 0 | 0 | 0 |
| 20 | 4½ | Urethane × 12 | Croton oil × 70 | 10/17 | 6, 14½, 20½, 21½, 23, 28½, 29½, 32½, 32½, 33½ | 20 | 9 | 3 |
| 20 | 4 | Ethylene glycol × 12 | „ „ × 70 | 1/19 | 32½ | 3 | 1 | 0 |
| 20 | 4 | Urethane × 12 | Liquid paraffin × 70 | 0/17 | — | 0 | 9 | 1 |
| 20 | 4 | Ethylene glycol × 12 | „ „ × 70 | 0/20 | — | 0 | 1 | 0 |
| 20 | 3½ | Urethane × 86 | — | 0/20 | — | 0 | 14 | 6 |
| 15 | 3½ | Ethylene glycol × 86 | — | 0/15 | — | 0 | 0 | 0 |

* Survivors : survivors after 6 weeks of croton oil treatment (i.e. the time when the first papilloma appeared in the whole series).

† Time from commencement of secondary treatment.

(3) Urethane alone, applied twice weekly for 43 weeks, did not induce a single papilloma.

(4) Two papillomas (1/18 and 1/19 survivors, respectively) developed in control groups receiving croton oil without previous urethane treatment.

(5) The incidence of lung adenomas was higher among mice receiving 12 or more applications of urethane than in control groups, but was not significantly higher in the group receiving a single painting of urethane.

(6) Lesions of the liver, resembling hepatomas to the naked eye, but proving histologically to be haemorrhages with necrosis of liver parenchyma, were present among the urethane treated animals, and especially frequently among those receiving continuous urethane treatment, but were absent among those not receiving urethane.

### DISCUSSION.

These results support the findings of Salaman and Roe (1953) that urethane, itself non-carcinogenic for mouse skin, can induce the initiating phase of carcinogenesis in that tissue. This experimental confirmation of the principle, predicted on the basis of the two-stage mechanism of carcinogenesis (Berenblum, 1952), that tumour production should theoretically be possible by the successive actions of two non-carcinogenic agents—a pure initiator followed by a pure promoter—thus adds to the validity of the two-stage hypothesis. The results are also of technical value for future experiments, since the use of a substance which has an initiating action free from a promoting action has obvious advantages over that of a complete carcinogen in which the promoting action has to be artificially repressed by very short action.

The present results differ in certain respects from those reported by Roe and Salaman (1954), a *single* dose of urethane being found ineffective in our experiments but effective in theirs. Differences in technique were probably responsible for this, e.g. the small area of treated skin (1 sq. cm.) instead of the whole skin of the back, the different strains of mice used, and the fact that the concentrations of reagents and the solvents were not the same.

A surprising feature of the present results was the remarkably long average latent period for tumour induction, compared to that observed when 9 : 10-dimethyl-1 : 2-benzanthracene or one of the other polycyclic hydrocarbons was used as initiator (Berenblum and Shubik, 1947). In the experiments with urethane and croton oil by Salaman and Roe (1953), the latent period was intermediate between the two. No explanation for these divergent results can be offered at present, though it should be pointed out that even with dimethylbenzanthracene as initiator (followed by standard croton oil treatment), different average latent periods have been observed by us in apparently identical experiments carried out on different occasions.

The development of lung adenomas following applications of urethane to the skin (also observed by Salaman and Roe, 1953) emphasize the striking sensitivity of the lung to this agent. [The minimal effective dose by the intraperitoneal route is about 20 mg. (Henshaw and Meyer, 1944)]. The lesions of the liver in urethane treated mice, which appeared by naked eye to resemble hepatomas, proved histologically to consist of extensive haemorrhages with necrosis, *but without any evidence of neoplasia*, contrary to the reports of Salaman and Roe (1953). Damage to the endothelial lining of blood vessels in the liver, with resulting hæmorrhage, caused by urethane administration, was described by Doljanski and Rosin (1944).

SUMMARY.

The previously reported observation that ethyl carbamate (urethane), though itself non-carcinogenic for mouse skin, is able to induce in that tissue the initiating phase of carcinogenesis, has been confirmed.

The expenses of this research were partly defrayed out of a generous grant from the Joseph and Helen Yeamans Levy Foundation.

### REFERENCES.

BERENBLUM, I.—(1952) *Clinical Problems in Cancer Research*, Sloan-Kettering Institute Seminar 1948–1949, 125.

*Idem* AND SHUBIK, P.—(1947) *Brit. J. Cancer*, **1**, 383.

DOLJANSKI, L. AND ROSIN, A.—(1944) *Amer. J. Path.*, **20**, 945.

GRAFFI, A., VLAMYNCK, E., HOFFMANN, F. and SCHULZ, I.—(1935) *Arch. Geschwülstforsch.* **5**, 110.

HENSHAW, P. S. AND MEYER, H. L.—(1944) *J. nat. Cancer Inst.*, **4**, 523.

ROE, F. J. C. AND SALAMAN, M. H.—(1954) *Brit. J. Cancer*, **8**, 666.

SALAMAN, M. H. AND ROE, F. J. C.—(1953) *Ibid.*, **7**, 472.